# Factors associated with tuberculosis treatment outcomes among tuberculosis patients attending tuberculosis treatment centres in 2016-2017 in Mogadishu, Somalia

**DOI:** 10.11604/pamj.2017.28.197.13439

**Published:** 2017-11-02

**Authors:** Marian Khalif Ali, Simon Karanja, Mohammed Karama

**Affiliations:** 1 Jomo Kenyatta University of Agriculture and Technology, Nairobi, Kenya; 2 Kenya Medical Research Institute, Nairobi, Kenya

**Keywords:** Tuberculosis in Somalia, tuberculosis treatment outcomes, treatment centers

## Abstract

**Introduction:**

World Health Organization (WHO) reported that tuberculosis (TB) was a major health problem and the second leading cause of mortality globally. An estimated 1.8 million TB deaths were reported in 2015. In Somalia, the average TB incidence was 274 cases per 100,000 people in 2014; prevalence was 513 per 100,000 population; and mortality rate excluding human immune deficiency virus (HIV)/TB co-infection was 64/100,000. In addition, the prevalence rates of multi-drug resistant (MDR)-TB are still high, 5.2% among new cases and 40.7% for retreatment cases. The objective of this study was to determine individual and institutional level factors associated with TB treatment outcomes (TB-TOs) among patients attending TBTCs in Mogadishu.

**Methods:**

The study design was cross-sectional, using quantitative and qualitative methods. Data was collected using interviewer administered semi-structured questionnaires and key in-depth interviews in 2016/2017. Qualitative data was coded using NVIVO8 and quantitative data analyzed using descriptive and inferential statistics at 95% confidence interval using SPSS20 software.

**Results:**

The study used a sample of 385 TB patients. There were 315(81.8%) successful TB-TOs. Individual level factors-marital status, education level, HIV status, treatment category and knowledge on TB influenced TB-TOs (p-value < 0.05). Being married, educated, HIV-negative, new treatment case and knowledgeable on TB increased odds of successful TB-TOs (OR > 0, p value < 0.05) compared to other patients. TBTCs factors did not influence TB-TOs (p-value > 0.05).

**Conclusion:**

TB-TOs were mainly affected by patient individual factors. There was need for patient education on TB management and treatment; and improved patient-health provider relationship.

## Introduction

TB is an infectious disease that commonly affects the lungs and is caused by bacteria-*Mycobacterium tuberculosis* [1]. Most infections with the bacteria do not cause TB disease and 90-95% of infections remain asymptomatic [2]. TB can cause infection in persons with impaired immunity [3]. Most infections do not have symptoms, known as latent TB, where in about 10% of these latent infections can develop disease in lifetime which can kill about half of those infected [4]. The symptoms of active pulmonary TB disease are coughing, with sputum or blood, chest pains, fever, weight loss and night sweats [5]. TB is transmitted through droplets from an infected person with active pulmonary disease released in the air through cough, sneeze or talking and then inhaled by another person [6]. TB can also spread through ingesting infected milk or meat (bovine TB) [7]. Medical evaluation of TB includes medical history of exposure, infection and other risk factors like HIV infection; physical examination to assess patient's general health to inform treatment plan; chest x-ray to detect chest abnormalities; and microbiological tests using samples of sputum [8]. Tuberculosis treatment aims to cure TB patients, prevent deaths from TB and to stop transmission of mycobacterium TB from the infected to the host community [9]. TB Treatment can be challenging for patients as it requires taking multiple drugs for at least 6 months [10]. The standard TB treatment regimen consists of an intensive phase that lasts 2 months involving 4 drugs (isoniazid, rifampicin, pyrazinamide and ethambutol) to rapidly kill the Mycobacterium tuberculosis [11]; and a continuation phase that last up to 4 months involving 2 drugs (isoniazid, rifampicin) that eliminate the remaining bacilli and prevent relapse [10]. WHO set the global target rate for a successful treatment outcome at 85% [12] and classified treatment outcomes as cured, treatment completed, treatment failed, died, lost to follow-up, not evaluated and treatment success including sum of cured and treatment completed. The treatment outcomes are influenced by socio-demographic and socio economic factors [13]; nutrition [14]; HIV [15]; MDR TB [5]; and strategies for TB management including DOTS.

TB remains among major health problems and leading global causes of mortality [16]. In 2015, there were an estimated 10.4 million new (incident) TB cases worldwide. The rate of decline in TB incidence remained at 1.5% from 2014 to 2015 [17]. United Nations through the WHO End TB Strategy in 2014 committed to 90% reduction in TB deaths and an 80% reduction in the TB incidence rate by 2030 [17]. TB accounted for 2.0% of all disability adjusted life years (DALYs) worldwide in 2010 [18]. Number of TB deaths fell by 22% between 2000 and 2015 [17], however TB remained one of the leading causes of deaths worldwide [16]. There were an estimated 480 000 new cases of MDR-TB and an additional 100 000 people with rifampicin-resistant TB (RR-TB) who were also newly eligible for MDR-TB treatment in 2015 [17]. Globally, 3.5% of new and 20.5% of previously treated TB cases was estimated to have had MDR-TB [16]. Over two thirds of the global TB burden is reported in Africa and Asia, where India, Indonesia and China account for the highest number of TB cases amounting to 43% of the global burden [2]. African region has had the highest TB prevalence, mortality [16] and burden [17]. In 2014, African region had approximately 28% of the world's cases and the most severe burden of TB relative to population: 281 cases for every 100 000 people more than double the global average of 133 [19]. HIV infection is the most important single predictor of TB incidence across the African continent [20]. The burden of TB in Africa was 34.2 million, that is, 2.2% of total of the world DALYS [21]. The poorest and socially excluded groups often carry the largest burden of TB [2]. Household costs for TB care in Africa accounted for almost one fifth of their annual income and are a barrier to access care particularly among the poor [22].

In Uganda, 70% of the cost of TB treatment is borne by patients and their families [22]. Somalia is the one of the most violent and poorest countries in the world with one of highest incidence rate of TB in the world [23]. TB is among major health burdens and major cause of morbidity and mortality in Somalia [24]. TB deaths in Somalia reached 6,458 or 5.03% of total deaths [25]. The age adjusted death rate is 123.01 per 100,000 of population; which ranks number four in the world making TB one of the leading cause of morbidity and mortality among the adult population, contributing to significant loss in work productivity and increased household expenses in support of affected member of the household during its long treatment [26]. The epidemiology of TB in Somalia is similar to other developing countries where the disease is related with widespread poverty, poor living conditions and reduced immune state especially those living with HIV and AIDS [26]. The 15-49 years age group is largely affected [24]. Estimated TB incidence and prevalence was 274 per 100,000 and 513 per 100,000 populations respectively [24]. The incidence of sputum smear positive cases was 160 per 100 000 population [16]. The estimated prevalence of MDR-TB is 5.2% among new cases and 40.8% among retreatment cases [27]. Despite the availability of free TB treatment in TB centers in Somalia, the prevalence rates of TB and MDR-TB still remain high. Previous studies has shown lower cure rates and higher mortality and re infection rate in HIV/TB co infected patients [28]. The levels of MDR-TB in Somalia are among the highest in the Eastern Mediterranean and African region [5]. Treatment of MRD-TB usually requires prolonged chemotherapy with highly toxic second-line drugs [29]. Although MDR-TB treatment was started in some regions of Somalia, Banadir and other regions are still suffering from lack of anti MDR TB drugs [5]. Knowledge of the real extent of TB and HIV co infection in Somalia is limited because of incomplete surveillance data.

## Methods

This study adopted cross-sectional design and used quantitative and qualitative methods. The study was carried out in all (seven) public TB management units (TB centres) in Mogadishu namely Manhal, Ayan, Mercy, Muslim Aid, Dharkenley, Sacid and Finsom TB centers. The study sample size was 385 TB patients attending the TB centres in Mogadishu determined using Cochran's formula [30] at 95% confidence level. The TB patients were randomly and proportionately sampled using stratified and simple random sampling techniques to complete researcher administered semi-structured questionnaires. From each TB center, at least one health worker was purposively selected and interviewed as key informants. Ethical approval to conduct research was sought from the university and Ministry of Health Somalia, Federal Republic of Somalia. Permission to conduct the study was sought from hospital authorities and consent from TB patients with confirmation of confidentiality. Quality control measures were employed including pre-test, completeness, validity and reliability checks. Validity of data was ensured through scrutiny for outliers and inconsistency in data collected.Qualitative data was coded through creation of categories and themes suing NVIVO8. Quantitative data analysis involved both descriptive and inferential statistics using SPSS version 20. Relationship between the independent variables and the dependent variable was established using Chi-square tests of association. Logistic regression was used to estimate Odds Ratio (OR) as a measure of association. Statistical significance was checked using 95% confidence interval and p-value of < 0.05 was considered significant. Findings were presented in form of text, tables and graphically.

## Results

**Individual characteristics of TB patients attending TBTCs**: The study sample was 385 TB patients from the seven TBTCs. The sample TB patients were mainly male 256(66.5%); with mean age of 32.2 years; married 199(51.7%); had Madrassa 128(33.2%) and primary education 83(21.6%); with consistent sources of income 139(36.1%); and monthly income of less than or equal to 200USD 283(75.3%) (The TB patients' households had a mean number of 6 persons. The mean number of TB patients' children was 3 (Table 1). The 385 TB patients had been on treatment for a mean of 4.01 months with 335(87%) being on medication for more than two months (Most 315(81.8%) patients were new cases. A great proportion of 375(97.4%) TB patients were HIV negative (Table 1).

**Table 1:** Individual factors of TB patients attending TBTCs-(a)

Variable	Unit	Number	Percentage	c2	df	P value
Gender	Male	256	66.5	39.520	1	<0.001
	Female	129	33.5			
Age groups	18-27%	175	45.5	32.592	6	0.004
	28-37%	105	27.3			
	38-47%	53	13.8			
	48-57%	26	6.8			
	58-67%	19	4.9			
	68-77%	5	1.3			
	78-87%	2	.5			
Marital status	Married	199	51.7	30.144	3	0.041
	Unmarried	135	35.1			
	Divorced	34	8.8			
	Widowed	17	4.4			
Level of education completed	None	110	28.6	17.512	3	0.031
	Madrassa	128	33.2			
	Primary/ Elementary	83	21.6			
	Secondary	64	16.6			
Current occupation	Government employee	30	7.8	14.449	1	<0.001
	Un-employed	108	28.1			
	Housewife	70	18.2			
	Business	27	7.0			
	Casual employee	95	24.7			
	Firm employee	4	1.0			
	Student	51	13.2			
Number of children	0	127	33.0	19.983	3	<0.001
	1-3%	117	30.4			
	4-6%	101	26.2			
	>6	40	10.4			
Household income per month in USD	<201	290	75.3	39.249	2	<0.001
	201-400%	78	20.3			
	>400	17	4.4			
Number of persons currently in patients' households	<6	190	49.4	29.678	2	0.027
	6-10%	175	45.5			
	>10	20	5.2			
Current month of TB treatment	<=2	50	13.0	42.114	1	<0.001
	>2	335	87.0			
Treatment category	New case	315	81.8	31.105	1	<0.001
	Re-treatment cases	70	18.2			
HIV status	Positive	10	2.6	69.110	1	<0.001
	Negative	375	97.4			

USD- USA Dollar; c2- Chi square; df- degrees of freedom; P value- Level of significance

**Knowledge on TB among TB patients attending TBTCs**: Eighty seven percent of TB patients were aware of TB before being diagnosed. The percent of patients informed by family, friends, neighbours or colleagues varied significantly (P < 0.001). A section of 22.9% patients were aware of the cause of TB (P < 0.001) while 54.5% patients were aware of the common signs and symptoms of TB (P = 0.060). Sixty six percent of the patients were aware that TB could be transmitted and prevented (P < 0.001). Proportions of 60.5% and 52% patients were aware of ways in which TB could be transmitted (P < 0.001) and ways in which TB could be prevented (P = 0.007). Ninety four percent of TB patients were aware that TB could be treated and cured (P < 0.001) and 91.9% patients were aware of how TB could be treated and cured (P < 0.001). Generally, a section of 8.1% TB patients had full knowledge on cause; signs and symptoms; possibility and ways of transmission; possibility and ways of prevention; and possibility and ways of treatment/ cure of TB (P < 0.001) (Table 2).

**Table 2:** Individual factors of TB patients attending TBTCs (b)

Variable	Unit	Number	Percentage	c2	df	P value
Heard about TB	Yes	335	87.0	42.110	1	<0.001
	No	50	13.0			
Cause of TB	Don't know	297	77.1	22.595	1	<0.001
	Know	88	22.9			
Common signs and symptoms of TB	Don't know	175	45.5	7.691	1	0.060
	Know	210	54.5			
Possibility of TB transmission	Don't know	130	33.8	38.975	1	<0.001
	Know	255	66.2			
Ways of TB transmission	Don't know	152	39.5	33.913	1	<0.001
	Know	233	60.5			
Possibility to prevent TB	Don't know	158	41.0	38.975	1	<0.001
	Know	227	59.0			
Ways of preventing TB	Don't know	268	69.6	0.814	1	0.007
	Know	117	30.4			
Existence of TB treatment and cure	Don't know	23	6.0	59.551	1	<0.001
	Know	362	94.0			
Ways of TB treatment	Don't know	43	11.2	50.772	1	<0.001
	Know	342	88.8			

c2- Chi square; df- degrees of freedom; P value- Level of significance

**Attitude of TB patients attending TBTCs**: Patients' attitude on TB was assessed using adherence to dose, perception on seriousness of TB and having talked to someone after diagnosis. A proportion of 285(74%) patients was significantly not positive about TB (P < 0.001). A section of 189(49.1%) TB patients did not talk to anyone about TB after they were diagnosed (P = 0.618). The rest mainly spoke to spouses 59(15.3%), parents 51(13.2%), medical workers 45(11.7%) and close friends 40(10.4%). A section of 90.9% of the TB patients adhered to TB dose (P < 0.001). The TB patients had missed doses for an average of 2.2 times due to depleted drugs; inaccessible TBTCs; and forgetfulness. A proportion of 169(43.9%) patients did not perceive TB is a serious disease unlike 152(39.5%) who perceived it was a serious disease (P = 0.016) (Table 3).

**Table 3:** Individual factors of TB patients attending TBTCs (c)

Variable	Unit	Number	Percentage	c2	df	P value
Attitude	Not positive	285	74.0	17.718	1	<0.001
	Somehow positive	100	26.0			
Talked about TB after diagnosis	No	189	49.1	0.249	1	0.618
	Yes	196	50.9			
Adherence to dose	Adhered	350	90.9	55.329	1	<0.001
	Not adhered	35	9.1			
Perception on seriousness of TB	Very serious	152	39.5	15.311	2	0.016
	Somewhat serious	64	16.6			
	Not very serious	169	43.9			

c2- Chi square; df- degrees of freedom; P value- Level of significance

**Institutional factors of TBTCs**: The TBTCs were moderately accessible to 225(58.4%) patients based on time taken and means of transport to the health facility (P < 0.001). All seven TBTCs were open from six in the morning to one past midday from Saturday to Thursday; and medicine was issued free of charge. However, 75(19.5%) and 77(20%) TB patients were not aware of days and time TBTCs were open respectively (P < 0.001). A proportion of 8(2.1%) patients reported not to have received the medicine free of charge (X^2^ = 16.133; df = 1; P = 0.018) (Table 4). DOT was applied by 10(2.6%) patients (P < 0.001). A proportion of 152(39.5%) TB patients received medicine every morning; and every three days each (P = 0.027). A section of 15(3.9%) patients were observed as they took medicine by health worker 8(2.1%), spouse 6(1.6%), relative 2(0.5%) and friend 1(0.3%) (P < 0.001). KII1 reported- *"DOTS not implemented fully in all facilities because of challenges of having the patient daily"* (Table 4). A section of 253(65.7%) patients had treatment supporters including relatives 124(32.2%), spouses 116(30.1%) and friends 13(3.4%) (P < 0.001). Health care staff also reported - *"Most facilities have established treatment supporters for patients"* (Table 4). A group of 253(62.6%) patients received nutritional support in form of maize and cooking oil and shared with family members (P < 0.001) (Table 4). Proportions of 143(37.1%) and 24(6.2%) patients were trained on TB (P < 0.029) and received health educational materials (P < 0.001) on TB from the TBTCs. Health staff service delivery to TB patients had a mean score of 5.43 where 1 was worst and 10 was best (X^2^ = 10.503; df = 5; P <0.001) (Table 4).

**Table 4:** Health facility factors of TB patients attending TBTCs

Variable	Unit	Number	Percentage	c2	df	P value
Physical accessibility of TBTCs	Hardly accessible	68	17.7	16.851	2	<0.001
	Moderately accessible	225	58.4			
	Easily accessible	92	23.9			
Frequency of receiving anti-TB medicines	Every morning	152	39.5	14.843	2	0.027
	Every 3 days	152	39.5			
	Weekly	81	21.0			
Observation when taking anti-TB drugs	Not observed	370	96.1	39.251	1	<0.001
	Observed	15	3.9			
Treatment supporter	Present	253	65.7	75.820	2	<0.001
	Absent	132	34.3			
Nutritional support	Given	241	62.6	48.835	3	<0.001
	Not given	144	37.4			
TB awareness/training	Not trained	242	62.9	55.329	4	0.029
	Trained	143	37.1			
Health educational materials for reading	Given	24	6.2	58.877	5	<0.001
	Not given	361	93.8			

**TB treatment outcome of TB patients attending TBTCs**: Treatment outcomes were successful among 315(81.8%) TB patients (P < 0.001). Specific successful treatment outcomes included cured 237(61.6%) and treatment completed 78(20.3%). Specific unsuccessful treatment outcome included treatment failed 26(6.8%), defaulters 24(6.2%), transferred 11(2.9%) and died 9(2.3%) (P = 0.043).

**Individual factors associated with treatment outcome of TB patients**: Multivariate analysis indicated that marital status, education level, HIV status and treatment category influenced treatment outcome. Married patients were more likely to have a successful treatment outcome (OR .3, 95% CI .1 to .6) as compared to the unmarried patients. Illiterate patients, patients who had attended madrassa and elementary education were less likely ((OR 4.1, 95% CI 1 to 15.9) (OR 4.5, 95% CI 1.2 to 17) (OR 5.9, 95% CI 1.6 to 21.8) respectively) to achieve successful treatment outcome compared to patients who had secondary education. Being HIV positive lowered the chances of successful treatment outcome (OR 4.4, 95% CI 1.1 to 17.7) compared to the HIV negative patients. New TB treatment cases were more likely to have successful treatment outcome (OR 5.2, 95% CI 2.9 to 9.2) as compared to re-treatment cases (Table 5). Patients with moderate knowledge on TB were less likely to achieve successful treatment outcome (OR 2.4, 95% CI 1 to 5.6) compared to those with knowledge (Table 6). TB patients' attitude and institutional factors did not significantly influence treatment outcome (Table 5).

**Table 5:** Individual factors associated with treatment outcome of TB patients attending TBTCs (a)

Variable	Treatment outcome		P-value	Multivariate OR(95% CI)	P-value	Bivariate OR(95% CI)
	U	S				
**Gender**						
Male	47	209	0.495	1.256(0.7-2.4)	0.899	0.965(0.6-1.7)
Female	23	106	Referent	Referent	Referent	Referent
**Age groups**						
18-27	24	151	0.524	0.357(0.02-8.5)	0.199	6.292(0.4-104)
28-37	25	80	0.769	0.625(0.03-14.3)	0.417	3.2(0.2-54)
38-47	12	41	0.694	0.529(0.02-12.3)	0.397	3.417(0.2-58.8)
48-57	2	24	0.371	0.206(0.01-6.6)	0.119	12(0.5-273)
58-67	4	15	0.702	0.521(0.02-14.8)	0.385	3.75(0.2-74.1)
68-77	2	3	0.904	1.255(0.03-50.97)	0.810	1.5(0.1-40.6)
78-87	1	1	Referent	Referent	Referent	Referent
**Marital status**						
Married	29	170	0.001	0.304(0.1-0.6)	0.059	1.658(0.98-2.8)
Unmarried	41	145	Referent	Referent	Referent	Referent
**Number of children**						
0	20	107	0.543	0.648(0.2-2.6)	0.616	0.764(0.3-2.2)
1-3	18	99	0.859	0.897(0.3-2.96)	0.657	0.786(0.3-2.3)
4-6	27	74	0.220	2.017(0.7-6.02)	0.076	0.392(0.1-1.1)
>6	5	35	Referent	Referent	Referent	Referent
**Educational level**						
Illiterate	20	90	0.044	4.073(1.0-15.9)	0.019	0.221(0.06-.8)
Madrassa	27	101	0.025	4.538(1.2-16.96)	0.007	0.184(0.05-.6)
Elementary	20	63	0.008	5.855(1.6-21.8)	0.004	0.155(0.04-.55)
Secondary	3	61	Referent	Referent	Referent	Referent
**Employment status**						
Employed	46	200	0.481	1.253(0.7-2.4)	0.726	0.907(0.5-1.6)
Un-employed	24	115	Referent	Referent	Referent	Referent
**House hold income per month (USD)**						
<201	56	234	0.886	0.885(0.2-4.7)	0.446	0.557(0.1-2.5)
201-400	12	66	0.673	0.684(0.1-3.99)	0.704	0.733(0.1-3.6)
>400	2	15	Referent	Referent	Referent	Referent
**Treatment category**						
New cases	40	275	0.001	0.16(0.08-0.31)	0.001	5.16(2.9-9.2)
Re-treatment	30	40	Referent	Referent	Referent	Referent
**HIV status**						
Positive	6	4	0.035	4.426(1.1-17.7)	0.003	0.137(0.038-0.5)
Negative	64	311	Referent	Referent	Referent	Referent

U- Unsuccessful; S- Successful; OR- Odds ratio; CI- Confidence interval

**Table 6:** Individual factors associated with treatment outcome of TB patients attending TBTCs (b)

Variable	Treatment outcome		P-value	Bivariate OR(95% CI)
	U	S		
**Knowledge on causes, symptoms, transmission, prevention and treatment of TB**				
No knowledge	2	6	0.821	1.227(0.2-7.3)
Low knowledge	25	90	0.396	1.472(0.6-3.6)
Moderate knowledge	34	197	0.048	2.37(1-5.6)
Knowledgeable	9	22	Referent	Referent

U- Unsuccessful; S- Successful; OR- Odds ratio; CI- Confidence interval

## Discussion

The study established that most treatment outcomes were successful in Mogadishu. However, this rate of successful treatment outcomes was slightly lower than that of studies conducted in southern Ethiopia [31], and Northern Ethiopia [32]. The rate of successful treatment outcome was also lower than the WHO set the global target rate for a successful treatment outcome [12]. This implied the need for measures to improve on successful treatment outcomes [33]. Such measures can be linked with those implemented in other regions including Finland [34], South Africa [35] and Southwestern Nigeria [36] that reported more unsuccessful treatment outcomes compared to Mogadishu. Findings from this supported literature on the fact that individual factors were associated with TB treatment outcome [1, 31, 37]. Marital status, education level and HIV status influenced treatment outcome. Married patients were more likely to have a successful treatment outcome as compared to the unmarried patients. This was similar to a study conducted in Turkey where married patients had higher successful treatment [37]. This was attributable to the fact that most patients had spouses as their treatment supporters [38]. Illiterate patients, patients who had attended madrassa and elementary education were less likely to achieve successful treatment outcome compared to patients who had secondary education. This corresponds to findings in study conducted in Turkey where patients with low education rate had a lower successful treatment outcome than those with more education [37]. Education level is perceived to reduce ignorance and increase knowledge on drugs management and consequences [39]. Being HIV positive lowered the chances of successful treatment outcome compared to the HIV negative patients. This was similar to findings in a study conducted in Ethiopia where HIV co-infected TB patients had a lower treatment success rate compared with the non-HIV infected patients [1]. In addition, previous studies has shown lower cure rates and higher mortality and re infection rate in HIV/TB co infected patients in Africa [28]; Finland [34]; Northern Ethiopia [32]; and South Africa [35]. The lower successful treatment can be attributed to the fact that as HIV infection progresses, CD4 cells count decline by about 50-80 cells/mm^3^ per year and the overall immune system of the person becomes less able to prevent the dissemination of M. tuberculosis in the body [40].

New TB treatment cases were more likely to have successful treatment outcome as compared to re-treatment cases. This finding was in agreement with that of a study conducted in Turkey [37]; Finland [34] and Southwestern Nigeria [36] where previous treatment history lowered chances of successful outcome. This supported the findings the levels of MDR-TB in Somalia are among the highest in the Eastern Mediterranean and African region" [5] and the higher prevalence of previously treated TB [27]. The retreatment cases are mostly found in areas with poor TB control programs. The retreatment cases are mostly due to improper use of antibiotics by TB patients which is a result of administration of wrong treatment regimens and poor adherence to anti-TB drugs [29]. Knowledge level on cause; signs and symptoms; possibility of transmission; possibility of prevention; and possibility of treatment/ cure of TB was higher in this study as compared to a study done in Indonesia [41]. Patients with moderate knowledge on TB were less likely to achieve successful treatment outcome compared to those with full knowledge. This finding was similar to that of a study conducted in China poor knowledge was perceived by the interviewees as an influencing factor [42]. TB patients should be informed about TB, preventive measures, diagnostic procedures and treatment modalities; and ounseled on possible adverse drug events in language they best understand to promote compliance [33]. TBTCs were less accessible compared to a study in northern Ethiopia [43]. Unlike in this study, various social and economic including poor healthcare provision services were found to be associated with negative treatment results [37]. Patients complained of disrespect and incomplete explanations on TB. Similarly, a study in Indonesia established that patients had experienced problems in communication with the hospital staff. According to these patients the doctors or nurses were sometimes unfriendly and could have little patience [41]. In China, unsuccessful outcomes were associated with lack of coordination of services by health staff [42]. Increase in health systems resources TB, improvements in facility maintenance, staff attitudes and communication, are likely to substantially improve TB patients' satisfaction which is highly attributed to successful outcomes [44]. Nutritional balance contributes to patient's treatment outcome [14] which was not the case in this study. This is because malnutrition results in delayed recovery as well as delay in sputum smear conversion of pulmonary TB patients [45]. Irrespective of DOTS strategy being implemented in most countries with high TB burden to increase case detection and treatment success rates and reducing incidence and morbidity rates among the population [46], its implementation has been very poor in Somalia. This could be attributed to treatment outcomes.

## Conclusion

**TB treatment outcomes**: Successful treatment outcomes were 81.8% among TB patients in Mogadishu. Specific successful treatment outcomes included cured (61.6%) and treatment completed (20.3%). Specific unsuccessful treatment outcome included treatment failed (6.8%), defaulters (6.2%), transferred (2.9%) and died (2.3%). There was need to work on measures aimed at improved TB treatment outcomes because the success rate was also lower than the WHO set the global target rate for a successful treatment outcome which is at 85% [12]. Individual factors influencing TB treatment outcomes: marital status, education level, HIV status, treatment category and knowledge on TB were the individual factors that influenced treatment outcome. TB-treatment centre factors influencing TB treatment outcomes: none of the institutional related factors including health facility accessibility, service delivery, nutrition, mode of medication, training and treatment supporter influenced treatment outcome.

### What is known about this topic

- Tuberculosis is a major health problem and the second leading cause of mortality globally;
- United Nations through the WHO End TB Strategy committed to 90% reduction in TB deaths and an 80% reduction in the TB incidence rate by year 2030;
- The prevalence rates of TB and multidrug-resistant (MDR)-TB still remain high in Somalia irrespective of free TB treatment in TB centres in Somalia.

### What this study adds

- Tuberculosis (TB) treatment outcomes among patients attending TB treatment centres in Mogadishu;
- Individual level factors associated with tuberculosis (TB) treatment outcomes among patients attending TB treatment centres in Mogadishu;
- Institutional level factors associated with tuberculosis (TB) treatment outcomes among patients attending TB treatment centres in Mogadishu.

## Competing interests

The authors declare no competing interest.
